# Investigation and optimization of PET-guided SPECT reconstructions for improved radionuclide therapy dosimetry estimates

**DOI:** 10.3389/fnume.2023.1124283

**Published:** 2023-06-21

**Authors:** Harry Marquis, Kathy P. Willowson, C. Ross Schmidtlein, Dale L. Bailey

**Affiliations:** ^1^ Department of Medical Physics, Memorial Sloan Kettering Cancer Center, New York, NY, United States; ^2^ Institute of Medical Physics, University of Sydney, Sydney, NSW, Australia; ^3^ Department of Nuclear Medicine, Royal North Shore Hospital, Sydney, NSW, Australia

**Keywords:** radionuclide therapy dosimetry, partial volume correction, theranostics, quantitative SPECT, SPECT reconstruction, SPECTRE reconstruction

## Abstract

**Introduction:**

To investigate and optimize the SPECTRE (Single Photon Emission Computed Theranostic REconstruction) reconstruction approach, using the hybrid kernelised expectation maximization (HKEM) algorithm implemented in the software for tomographic image reconstruction (STIR) software library, and to demonstrate the feasibility of performing algorithm exploration and optimization in 2D. Optimal SPECTRE parameters were investigated for the purpose of improving SPECT-based radionuclide therapy (RNT) dosimetry estimates.

**Materials and Methods:**

Using the NEMA IEC body phantom as the test object, SPECT data were simulated to model an early and late imaging time point following a typical therapeutic dose of 8 GBq of ^177^Lu. A theranostic ^68^Ga PET-prior was simulated for the SPECTRE reconstructions. The HKEM algorithm parameter space was investigated for SPECT-unique and PET-SPECT mutual features to characterize optimal SPECTRE parameters for the simulated data. Mean and maximum bias, coefficient of variation (COV %), recovery, SNR and root-mean-square error (RMSE) were used to facilitate comparisons between SPECTRE reconstructions and OSEM reconstructions with resolution modelling (OSEM_RM). 2D reconstructions were compared to those performed in 3D in order to evaluate the utility of accelerated algorithm optimization in 2D. Segmentation accuracy was evaluated using a 42% fixed threshold (FT) on the 3D reconstructed data.

**Results:**

SPECTRE parameters that demonstrated improved image quality and quantitative accuracy were determined through investigation of the HKEM algorithm parameter space. OSEM_RM and SPECTRE reconstructions performed in 2D and 3D were qualitatively and quantitatively similar, with SPECTRE showing an average reduction in background COV % by a factor of 2.7 and 3.3 for the 2D case and 3D case respectively. The 42% FT analysis produced an average % volume difference from ground truth of 158% and 26%, for the OSEM_RM and SPECTRE reconstructions, respectively.

**Conclusions:**

The SPECTRE reconstruction approach demonstrates significant potential for improved SPECT image quality, leading to more accurate RNT dosimetry estimates when conventional segmentation methods are used. Exploration and optimization of SPECTRE benefited from both fast reconstruction times afforded by first considering the 2D case. This is the first in-depth exploration of the SPECTRE reconstruction approach, and as such, it reveals several insights for reconstructing SPECT data using PET side information.

## Introduction

Single Photon Emission Computed Tomography (SPECT) is commonly used to evaluate the radiation dose delivered to target structures and normal organs in radionuclide therapy (RNT). Currently, SPECT imaging is hindered by poor spatial resolution, making accurate quantification of the dose delivered difficult to measure accurately (1). This has slowed the progress of the personalization of radionuclide therapies, where the dose is tailored to the individual, and where diagnostic PET imaging prior to therapy can be used to optimize the delivery of therapeutic radionuclides for the management and treatment of a range of cancers. Improved quantitative accuracy in SPECT imaging of RNT should lead to a better understanding of the radiobiological effects of targeted radionuclide therapies and may pave the way forward for improved personalization in RNT treatment planning.

Reconstruction of SPECT data acquired with ME collimators presents several difficulties, where the relatively high noise and poor spatial resolution (compared to PET) pose significant challenges for image reconstruction algorithms (2). The poor spatial resolution of SPECT and, to a lesser extent, PET manifests as so-called partial volume effects (PVEs), which result in the systematic underestimation of image-based activity measurements in objects smaller than 2–3 times the full-width at half-maximum (FWHM) of the imaging system, also known as the system point spread function (PSF) (3). Optimization of SPECT acquisition protocols and image reconstruction algorithms typically requires knowledge of the ground truth (GT) radioactivity concentration. Both physical experiments and simulated studies can be used to evaluate various aspects of SPECT acquisition protocols and image reconstruction algorithms. A common approach for reconstruction algorithm optimization is to perform physical phantom experiments with fillable compartments with known geometry and radioactivity concentrations (4–10). Such experiments are typically designed to model a particular cohort of patients, both in terms of physical structure and radioactivity uptake. Simulation of emission tomography (ET) data using digital phantoms is an alternative to conducting physical phantom experiments (11, 12), where complex radioactivity distributions can be readily modelled (13). The most common approach for simulating nuclear medicine imaging systems is the Monte Carlo (MC) method, which is a well-established technique capable of modelling the physical processes involved in PET and SPECT image acquisition (14–18). Monte Carlo methods are useful, for example, in estimating the scatter component of SPECT acquisitions in order to evaluate the accuracy of various scatter correction methods (19–24).

Routine use of MC methods for simulating ET data is hindered by the computational time and resources required to accurately simulate the number of detected events typically acquired in a routine clinical PET or SPECT scan (25). Alternatives to MC methods are so-called analytical methods, where the simulation of PET and SPECT data involves calculating the mean estimate of a sinogram bin based on a mathematical geometric ray-tracing model. Analytical methods have the advantage of being much faster than MC methods, meaning that multiple realizations can be produced more quickly (19, 26). Analytical methods are suitable for evaluating image reconstruction algorithms, testing novel image processing methods, evaluating image quality (e.g., noise, resolution, etc.), and investigating acquisition protocols and reconstruction settings (27–30). Several analytical PET simulators are available: ASIM (26), SMART (29), STIR (31), and PETSTEP (27), to name a few. The software for tomographic image reconstruction (STIR) library is an open-source image reconstruction toolkit written in C++ that provides classes and utilities for SPECT and PET image reconstruction, analytical simulations, image manipulation, and image analysis. A number of reconstruction algorithms are available in STIR, including analytic, iterative, and anatomically driven approaches. Most of STIRs’ user base is involved in the research, development, and optimization of PET reconstruction algorithms. Fuster et al. first introduced the SPECT-UB utilities into the STIR reconstruction library in 2013 (32) and showed that noise-free SPECT projections of a cylindrical phantom generated using the SPECT-UB utilities (*ProjMatrixByBinSPECTUB*) were similar to sinograms simulated using the SimSET MC code (33), suggesting that the SPECT-UB utilities may be useful for fast analytical simulations of SPECT projection data. The SPECT-UB utilities are capable of modelling attenuation [using a modified implementation of the Siddon algorithm (34)] and PSF contributions to the projection matrix (32, 35). Currently, STIR cannot model scatter in SPECT imaging, though it can correct for it if the scatter estimate (SE) is supplied. A survey of the literature suggests that the use of STIR for analytical simulations of SPECT projection data has not yet been investigated, despite its promise being suggested as early as 2013.

In previous work, we introduced the SPECTRE (Single Photon Emission Computed Theranostic Reconstruction) reconstruction approach to address the current limitations of SPECT imaging of therapeutic radionuclides (36). The SPECTRE reconstruction approach uses diagnostic PET images to guide reconstruction of the SPECT data in a theranostic setting. This novel approach to SPECT image reconstruction uses the hybrid kernelised expectation maximization algorithm (HKEM) implemented in STIR by Deidda et al. (12) and is, to the best of our knowledge, the first example of PET-guided SPECT reconstruction. This reconstruction approach demonstrated potential for improved SPECT resolution and image quality, ultimately leading to more accurate SPECT-based dosimetry estimates (36). Previous MR-guided PET reconstruction studies using the kernelised expectation maximisation algorithm have suggested that larger kernel windows can lead to PET-unique feature suppression (12, 37, 38), that is, features present in the data being reconstructed can be suppressed when those features are absent in the guiding modality. Due to the different characteristics of SPECT resolution and noise and the use of PET-images as the guiding modality, previous investigations looking at the HKEM algorithm applied to MR guided PET are not necessarily applicable to SPECTRE and thus warrant an in-depth and standalone investigation.

## Materials and methods

The investigation and optimization of the SPECTRE reconstruction approach using the HKEM algorithm are performed in three steps. In the first step, 2D simulated data is used to investigate the impact of a range of parameters on PET-SPECT mutual and SPECT-unique features. In the second step, the parameters for these 2D reconstructions are then optimised at two noise levels. In the third step, the reconstructions from 2D simulations were compared to those from 3D simulations to validate the use of 2D results for optimizing 3D reconstruction, and the 3D reconstructed images were then analysed to evaluate the impact of their improved image quality on accurate volume delineation and quantitative mean and maximum value recovery.

### SPECTRE image reconstruction

The HKEM algorithm, developed by Deidda et al. (12), is a basis-constrained version of the well-known Maximum Likelihood Expectation Maximization (MLEM) algorithm. The HKEM algorithm was originally implemented for PET image reconstruction using a MR prior, and in previous work, we extended its use to SPECT reconstruction guided by a PET-prior in a theranostic setting, which we termed SPECTRE (36). Briefly, the SPECTRE algorithm is given by:

(1) $$\alpha_{f}^{\left(n+1\right)}=\frac{\alpha_{f}^{\left(n\right)}}{\sum_{j}k_{\;p,f\;j}k_{s,f\;j}^{\left(n\right)}\sum_{i}p_{ij}}\sum_{j}k_{\;p,f\;j}k_{s,f\;j}^{\left(n\right)}\sum_{i}\frac{p_{ij}\;\;y_{i}}{\sum_{l}p_{il}\sum_{f}k_{\;p,fl}k_{s,fl}^{\left(n\right)}\alpha_{f}^{\left(n\right)}+s_{i}},$$

where $\alpha_{f}^{\left(n\right)}$ is a coefficient image at each location *f* and *y_i_* is the sinogram data. The basis for the constrained image is thus:

(2) $$x_{j}=\sum_{f=1}^{N_{j}}\alpha_{f}k_{s,fj}K_{\;p,fj},$$

where *k_p_* and *k_s_* are the basis kernels derived from a PET prior and the SPECT update image, respectively. The PET and SPECT kernels map the similarity between the local voxel index “*f*” and the supporting voxel index “*j*”, in the kernel window. The PET and SPECT kernels using the HKEM algorithm applied to the SPECTRE reconstruction approach are defined as (36):

(3) $$k_{\;pfj}=k_{p}(v_{f},v_{j})=\exp\left(-\frac{{\left\|v_{f}-v_{j}\right\|}^{2}}{2\alpha_{p}^{2}}\right)\;\exp\left(-\frac{{\left\|x_{f}-x_{j}\right\|}^{2}}{2\alpha_{dp}^{2}}\right)$$

and,

(4) $$k_{s\;f\;j}^{\left(n\right)}=k_{s}(z_{f}^{\left(n\right)},z_{j}^{\left(n\right)})=\exp\left(-\frac{{\left\|z_{f}^{\left(n\right)}-z_{j}^{\left(n\right)}\right\|}^{2}}{2\alpha_{s}^{2}}\right)\;\exp\left(-\frac{{\left\|x_{f}-x_{j}\right\|}^{2}}{2\alpha_{ds}^{2}}\right)$$

**Figure 1:**
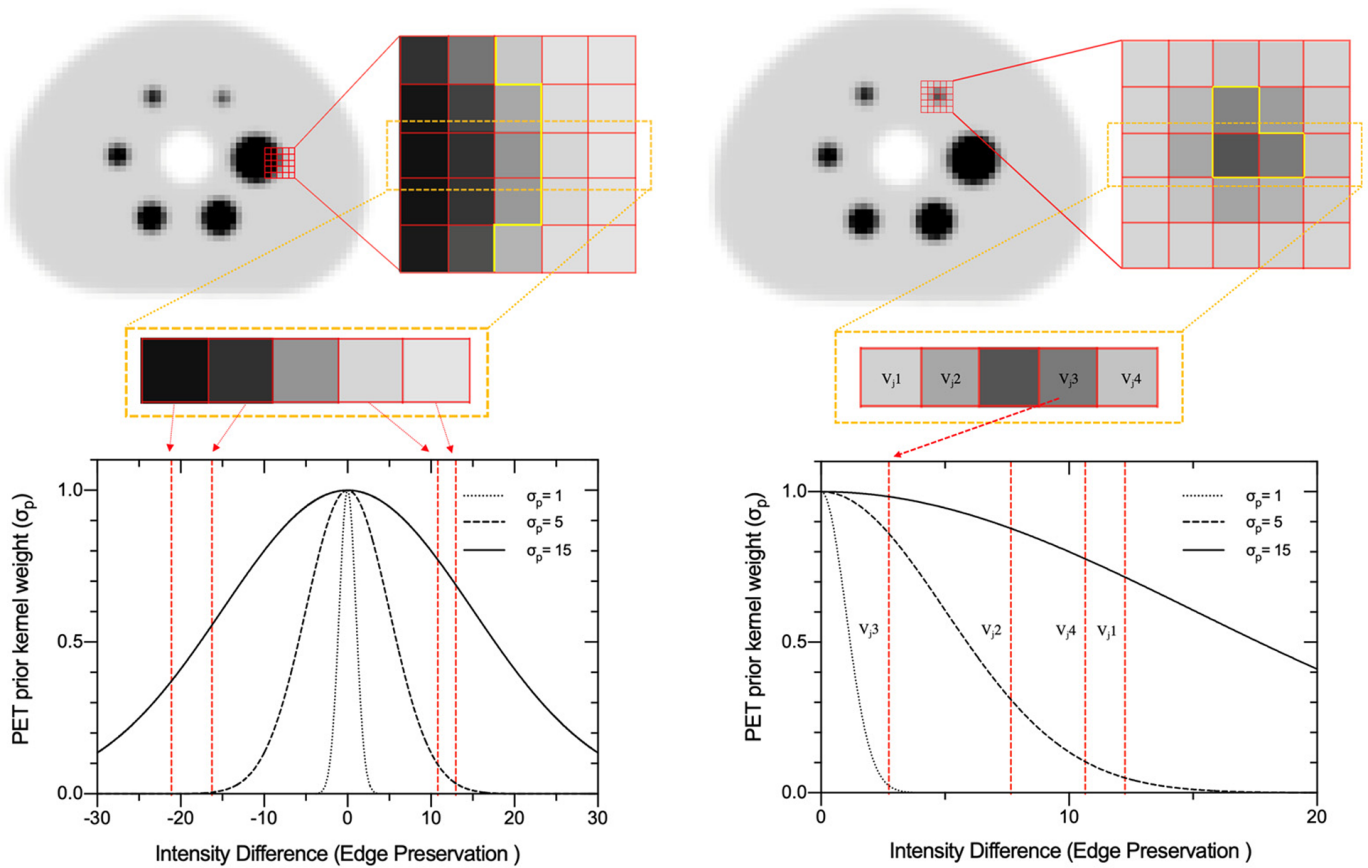
PET-prior intensity weights for different values of *σ*_p_ for a simulated PET image of the NEMA IEC body phantom. Left: 5 × 5 window placed on the edge of the largest sphere (37 mm diameter), where the centre row is extracted to demonstrate the support of *σ*_p_ values 1, 5, and 15 in SPECTRE reconstruction method. Right: a 5 × 5 window placed on the centre voxel of the smallest sphere (10 mm diameter) to demonstrate the impact of *σ*p on lower contrast structures.

The HKEM algorithm has five adjustable parameters that can be optimised in SPECTRE for a particular reconstruction task. These parameters are the PET-prior intensity weight (*σ*_p_), SPECT update-image intensity weight (*σ*_s_), Euclidean distance weights coming from the PET-prior and SPECT update-image (*σ*_dp_ and *σ*_ds_, respectively), and the size of the kernel search window (NN). The strength of the Gaussian-weighted intensity supports within the kernel window are set by parameters *σ*_p_ and *σ*_s_. These parameters control the degree of edge preservation coming from the voxel intensity differences in the PET-prior ($v_{f}$ and $v_{j}$) and SPECT update images (*z_f_* and *z_j_*), respectively. Figure 1 shows an example of edge preservation coming from the voxel intensity differences between *v_f_* and *v_j_*, when using a PET-prior simulated from an IEC phantom Digital Reference Object (DRO). The degree of edge preservation coming from the PET-prior is normalised to the standard deviation of the PET image. Large values of *σ*_p_ will impose similar weights on all voxels within the kernel window, which may result in oversmoothing across edges in the reconstructed image. Smaller *σ*_p_ values promote edges for voxels with small intensity differences, which may result in the promotion of edges coming from noise in the reconstructed PET image. On the other hand, the motivation for setting the edge preservation from the SPECT update image (*σ*_s_) is different when compared to *σ*_p_. For the case of *σ*_s_, the local voxel value (*z_f_*) in the SPECT update image (centre voxel in the kernel window) are normalised by the *σ*_s_ intensity weights for the supporting voxel (*z_j_*). This means that *σ*_s_ weights the voxel neighbourhood in the kernel window allowing edge preservation coming from the SPECT update image to handle SPECT PVEs more efficiently than *σ*_p_ alone.

The degree of support (local voxel, *x_f_*, and supporting voxel *x_j_*), is controlled by the scaling parameters *σ*_dp_, and *σ*_ds_. These control the degree of edge preservation for *σ*_p_ and *σ*_s_ in terms of the Euclidean distance from the centre voxel within the kernel window. Smaller values impose greater weighting on voxels closer to the centre voxel, resulting in greater edge preservation. The radial Gaussian functions for the distance weights are normalised to the SPECT voxel size.

### Simulated SPECT data

**Figure 2:**
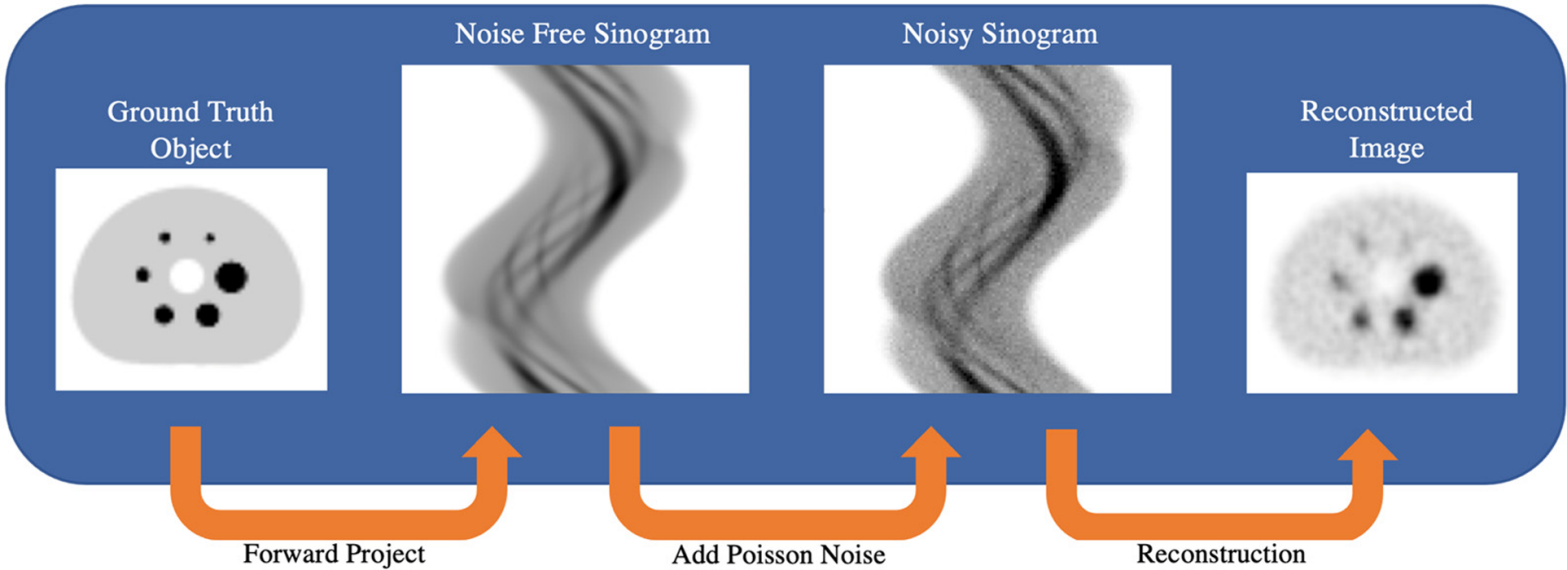
Schematic showing the process of simulating SPECT projection data in STIR. Ground truth DRO forward projection uses an analytically calculated collimator-detector response function (CDRF) to produce noise-free sinograms. Poisson noise is added to the simulated sinograms to reflect the number of counts that may be acquired under experimental and/or clinical imaging protocols.

The STIR simulated SPECT data aimed to model two noise levels from a NEMA IEC phantom study presented in previous work (36). Briefly, the phantom experiment had an 8.5:1 sphere-to-background ratio and was acquired on a dual-head Intevo 6 Siemens SPECT/CT system with Medium-Energy-Low-Penetration (MELP) collimators with an acquisition matrix size of 256 × 256 (resampled to 128 × 128), with 120 projections acquired over 360° (3° sampling) using continuous rotation mode and with body contouring on. The study was acquired with a 30 s per projection angle dwell time. Aliquot measurements determined that the spheres and background compartment had ^177^Lu radioactivity concentrations at scan time of approximately 2,784 kBq/ml and 317 kBq/ml, respectively. A ground-truth digital reference object (DRO) was generated by manually segmenting a CT image of the IEC phantom and assigning the measured activity concentrations to the sphere and background compartments. The DRO was resampled to the SPECT voxel size (4.8 mm cubic voxels) and co-registered with the SPECTRE reconstructed image of the experimentally acquired data presented in previous work (36).This was done so that realistic non-circular projection radii (from the experimental SPECT projection data) could be used in the simulation. The DRO was forward projected in STIR with a 2D and 3D PSF using an analytically determined ^177^Lu collimator-detector response function (CDRF) (39), and “full” attenuation modelling using an estimated µ-map (40). Poisson noise was added to the noise-free simulated projections based on an experimentally determined ^177^Lu system sensitivity of 12.2 cps/MBq per gamma camera. Two noise levels were generated, each reflecting the typical number of counts detected using a clinical acquisition protocol following a typical administered therapeutic activity of 8 GBq of ^177^Lu-(DOTA-Octreotate—“LUTATE”). The first set of SPECT data has counts typical of an early imaging time-point, and the other reflecting the typical number of counts acquired at a later imaging time point. The two noise levels are referred to as low-noise “PN1” (“*Poisson-Noise level 1*”) and high-noise “PN2” simulations, respectively. A simplified schematic of the simulation process is shown in Figure 2. The PN1 and PN2 simulated SPECT data were reconstructed in STIR on a 2016 MacBook Pro with a 3.3 GHz Intel Core i7 processor with 16 GB of RAM.

### Simulated PET prior(s)

**Figure 3:**
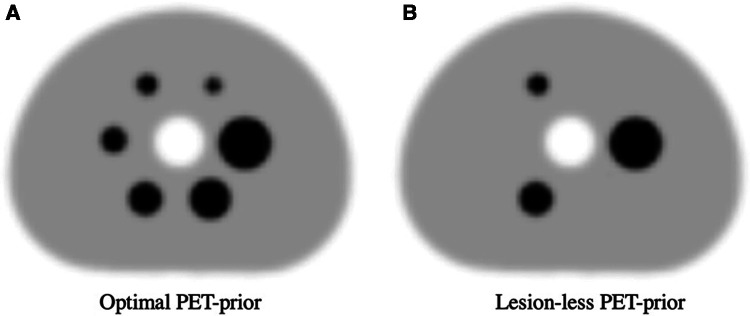
The two PET-priors used in the SPECTRE reconstructions. (**A**) Optimal PET-prior generated by applying a 7.5 mm FWHM Gaussian filter to the GT DRO, (**B**) “Lesion-less” PET-prior with the 28, 17, and 10 mm spheres removed.

The PET-priors were generated by applying a 7.5 mm FWHM Gaussian filter to the ground truth (GT) DRO to approximately model the global spatial resolution relative to clinical ^68^Ga PET images (2, 36). To simplify and focus the parameter optimization study, PET image noise, spatially variant PSF, and reconstruction-related artifacts were not modelled in the priors. While these PET image characteristics are expected to influence the SPECTRE reconstructed image, we decided that since this is the first in-depth exploration of the HKEM parameter space in the context of the SPECTRE reconstruction approach, adding further complexities such as PET image noise may complicate things unnecessarily. This should, however, be the subject of future work. Two PET-priors were investigated: one uses an “***optimal***” PET-prior that includes all regions of elevated activity, and the other uses a “***lesion-less***” PET-prior that is missing several regions of elevated activity (28, 17, and 10 mm spheres are missing, see Figure 3). Hence, the SPECTRE reconstructions with the lesion-less PET-prior have three PET-SPECT mutual features and three SPECT-unique features. The standard deviation in the optimal and lesion-less PET priors is 71.74 and 71.10, respectively.

### Regions of interest analysis

Circular ROIs and 3D VOIs were created to best match the known cross-sectional areas and spherical volumes of each sphere, with the number of voxels rounded down if the known cross-sectional area/volume was exceeded. This was done to ensure that the ROIs/VOIs were not larger than the ground truth object. For the background, the ROI/VOI were drawn freehand, avoiding potential spill-in/out from the spheres/phantom edge. The ROIs/VOIs were propagated to the slice(s) of each reconstructed image to obtain the mean, maximum, and standard deviation in each of the spheres and the background compartment. Image-based metrics used for the comparison are the contrast recovery coefficient (CRC) (41), sphere SNR (6, 42–46), and the root-mean-square error (RMSE). The CRC, SNR, and RMSE were calculated as follows:

(5) $$\mathrm{CRC}=\frac{\left(({\bar{x}}_{\mathrm{VOI}}/B_{\mathrm{VOI}})-1\right)}{\left((A_{\mathrm{sphere}}/A_{\mathrm{bkg}})-1\right)}$$

(6) $$\mathrm{SNR}={\bar{x}}_{\mathrm{VOI}}-B_{\mathrm{VOI}}/B\sigma_{\mathrm{VOI}}$$

(7) $$\mathrm{RMSE}=\sqrt{(\mathrm{CO}V^{2}RC^{2})+{\left(\mathrm{RC}-1\right)}^{2}}$$

where ${\bar{x}}_{\mathrm{VOI}}$ and $B_{\mathrm{VOI}}$ are the image-based radioactivity concentrations in the sphere and background compartments, respectively, and metrics *A*_sphere_ and *A*_bkg_ are the ground truth radioactivity concentrations (2,784 and 317 kBq/ml, respectively). B*σ*_VOI_ is the standard deviation in the background compartment, and COV and RC are the sphere coefficient of variation and mean sphere recovery coefficients, respectively. The RMSE was only calculated for the three largest spheres since the COV contains the standard sphere standard deviation, which may lead to erroneous conclusions when a small voxel sample size is used. The CRC, SNR, and RMSE for both SPECT-unique features (sphere diameters 28, 17, and 10 mm) and mutual PET-SPECT features (sphere diameters 37, 22, and 13 mm) were analysed to investigate the impact of each HKEM parameter. Where applicable, coefficient of variation, mean bias, and maximum bias were calculated for SPECTRE and OSEM_RM reconstructions using the three largest ROIs (background compartment, 37, and 28 mm sphere). The mean and maximum bias were calculated as follows:

(8) $$\mathrm{Mean}\;\mathrm{Bias}(\text{\%})=100\times \frac{\left({\bar{x}}_{\mathrm{VOI}}-A_{\mathrm{true}}\right)}{\left(A_{\mathrm{true}}\right)}$$

(9) $$\mathrm{Max}\;\mathrm{Bias}(\text{\%})=100\times \frac{\left({\bar{x}}_{\mathrm{VOI}\_\max}-A_{\mathrm{true}}\right)}{\left(A_{\mathrm{true}}\right)}$$

where ${\bar{x}}_{\mathrm{VOI}}$ and ${\bar{x}}_{\mathrm{VOI}\_\max}$ are the mean and maximum radioactivity concentrations in the VOI/ROI, and *A*_true_ is the ground truth radioactivity concentration, which for sphere bias and background bias are A_sphere_ (2784 kBq/ml) and *A*_bkg_ (317 kBq/ml), respectively.

### Investigation 1, 2D SPECTRE reconstructions with missing PET support

The first SPECTRE investigation was performed using the 2D PN1 simulated data and the “lesion-less” PET-prior (shown in Figure 3B) to evaluate the impact of the HKEM parameters on various image-quality metrics for PET-SPECT mutual features and SPECT-unique features. The HKEM algorithm reconstruction parameters for this investigation are summarised in Table 1 and were chosen based on a preliminary exploration of parameters that demonstrated a high degree of PET & SPECT update image support (low values of *σ*_s_, *σ*_p_, *σ*_dp_ and *σ*_ds_) to more relaxed support (larger values of *σ*_s_, *σ*_p_, *σ*_dp_ and *σ*_ds_). Kernel window sizes larger than NN = 7 were not investigated due to the impractical reconstruction times required when applied to 3D data. Each 2D SPECTRE reconstruction was performed for 20 iterations using 12 subsets, with full attenuation correction and 2D RM using an analytical ^177^Lu CDRF. The reconstructed images were converted from counts to units of kBq/ml.

**Table 1:** A summary of the HKEM parameters used in the PN1 2D SPECTRE reconstructions using the “lesion-less” PET-prior. All reconstructions used 20 iterations with 12 subsets.

Baseline HKEM parameters	Investigated parameters
*σ*_p_ = 1, *σ*_dp_ = *σ*_ds_ = 1, NN = 5	*σ*_s_ = 0.1, 1, 5
*σ*_s_ = 1, *σ*_dp_ = *σ*_ds_ = 1, NN = 5	*σ*_p_ = 0.1, 1, 5
*σ*_s_ = 1, *σ*_p_ = 1, NN = 5	*σ*_dp_ & *σ*_ds_ = 1, 3, 5
*σ*_s_ = 1, *σ*_p_ = 1, *σ*_dp_ = *σ*_ds_ = 1	NN = 3, 5, 7

### Investigation 2, OSEM_RM and SPECTRE reconstructions using the optimal PET-prior

Parameters from the “lesion-less” PET-prior investigation showing improved CRC, SNR, and RMSE in features supported by the PET image (37, 22, and 13 mm diameter) were used to reconstruct the **PN1** (low-noise) and **PN2** (high-noise) 2D data using OSEM with RM (OSEM_RM) and SPECTRE. The SPECTRE reconstructions used the “Optimal” PET-prior containing all six spheres, as shown in Figure 3A), and HKEM parameters *σ*_p_ = *σ*_s_ = 5, *σ*_dp_ = *σ*_ds_ = 5, NN = 5. SPECTRE and OSEM_RM reconstructions were performed for 5, 10, 15, and 20 full iterations using 12 subsets. The PN1 and PN2 data were also reconstructed using OSEM without RM (OSEM std) for 4 iterations using 12 subsets and with a post-reconstruction Gaussian filter of 8 mm FWHM applied. All OSEM and SPECTRE reconstructions used full attenuation correction.

### Investigation 3, 2D and 3D reconstruction validation using OSEM_RM and SPECTRE

To validate the 2D optimization approach, the 2D and 3D PN1 (low noise) simulated data were used to investigate the similarities between the 2D and 3D OSEM_RM and SPECTRE reconstructed images. HKEM parameters with the Optimal PET-prior were altered to explore the parameter space (HKEM parameters *σ*_p_ = 5, *σ*_s_ = 2, *σ*_dp_ = *σ*_ds_ = 15, NN = 5). SPECTRE and OSEM_RM reconstructions were performed in 2D and 3D from 4 to 40 full iterations in increments of 4 iterations using 12 subsets (a total of 10 quantitative reconstructions for each series). The 2D ROI and 3D VOI COV %, mean bias, maximum bias, and SNR for the 2D and 3D reconstructed images were measured. Estimating the absorbed dose to lesions following RNT not only relies on the quantitative accuracy of our SPECT-reconstructed images but also on the accuracy of our segmented volumes. To assess the impact of SPECTRE on segmentation, we compared SPECTRE to OSEM_RM using a 42% fixed threshold (FT) segmentation method (47). The 42% FT threshold method was applied to all six IEC phantom spheres in the ground truth. DRO, 3D OSEM_RM, and SPECTRE reconstructed images and the resulting segmented volumes were compared to the known sphere volumes.

## Results

The results follow the same ordering as presented in the methods.

### SPECTRE reconstructions with missing PET support

**Figure 4:**
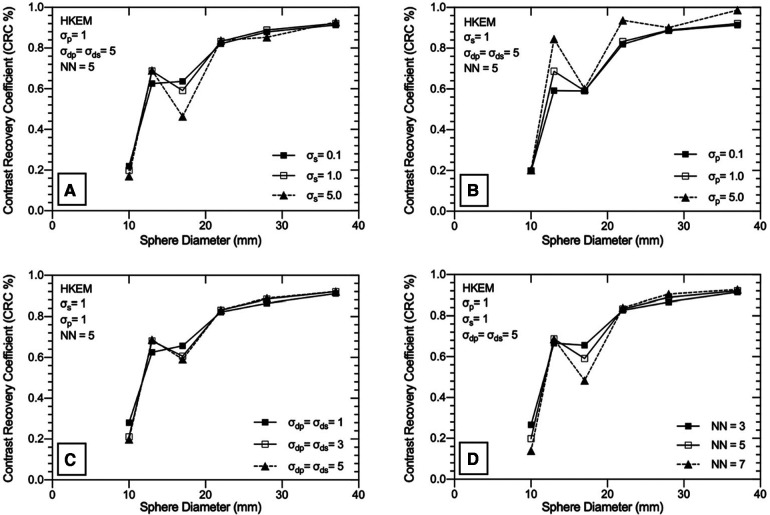
Exploration of the HKEM algorithm parameters using the “lesion-less” PET-prior in SPECTRE reconstructions of the PN1 2D simulated data. The CRC in each sphere is shown to investigate the impact of each parameter on PET-SPECT mutual and SPECT-unique features. (**A**) varying *σ*s parameter, (**B**) varying *σ*_p_ parameter, (**C**) varying *σ*_dp_ and *σ*_ds_ parameter, (**D**) varying NN parameter. Results are shown for the 20th iteration of the SPECTRE (HKEM) reconstructions.

**Figure 5:**
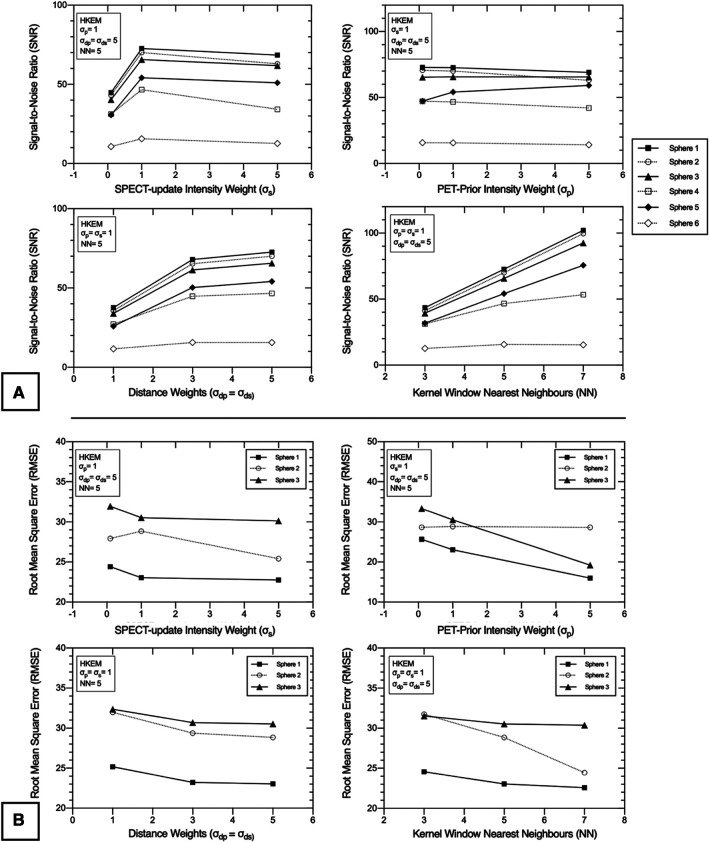
2D PN1 data was reconstructed with the SPECTRE reconstruction approach using the “lesion-less” PET-prior. Impact of HKEM parameters on the (**A**) signal-to-noise ratio (SNR) in all six IEC phantom spheres, and (**B**) root-mean-square-error (RMSE) in the three largest IEC phantom spheres, for each of the SPECTRE reconstructions. The three smallest spheres were not included in the RMSE analysis due to the small voxel sample size. The SNR and RMSE are shown for SPECTRE reconstructions at 20 full iterations. PET-SPECT mutual spheres are displayed as black/solid, and SPECT-unique spheres are displayed as unfilled/dashed.

Nine separate SPECTRE and a conventional OSEM reconstruction with resolution modelling (OSEM_RM, 20it12s) were used with the lesion-less data to measure the COV, CRC, SNR, and RMSE in Table 2, Figures 4, 5A,B, respectively.

**Table 2:** Coefficient of variation (COV %) in the background compartment for OSEM_RM and SPECTRE reconstructions using the “lesion-less” PET-prior. Note some redundancy due to the reuse of the baseline parameters (*σ*_p_ = *σ*_s_ = 1, *σ*_dp_ = *σ*_ds_ = 5 and NN = 5). BKG is short for “background”.

Parameter	Value	BKG COV %
*σ*_s_	0.1	15.9
	1	9.9
	5	10.6
*σ*_p_	0.1	9.8
	1	9.9
	5	11.1
*σ*_dp_ = *σ*_ds_	1	18.8
	3	10.5
	5	9.9
NN	3	16.4
	5	9.9
	7	7.1
OSEM_RM	–	29.2

In Table 2, the noise in the background compartment is shown to be highest for the OSEM_RM reconstruction (COV = 29.23%) and all SPECTRE reconstructions show improved noise characteristics with reduced BKG COV %. The CRC in each sphere for the investigated SPECTRE parameters using the lesion-less PET-prior is shown in Figure 4. SPECT update intensity weight (*σ*_s_) shows improved CRC in the two smallest SPECT-unique spheres (spheres 4 & 5, 17, and 10 mm diameters, respectively). Larger values of *σ*_s_ show improved CRC in all PET-SPECT mutual features. Using a PET-prior intensity weight (*σ*_p_) equal to 5 for PET-SPECT mutual features (spheres 1–3, diameters 37, 28, and 22 mm), produced significantly higher CRC over *σ*_p_ values of 0.1 and 1. For SPECT-unique features, *σ*_p_ does not appear to significantly influence the CRC. Euclidean distance weights (*σ*_dp_ and *σ*_ds_) showed improved CRC for SPECT-unique features. The opposite is true for the PET-SPECT mutual features, where more relaxed distance weights (higher values of *σ*_dp_ and *σ*_ds_) have a slightly higher CRC. The CRC for PET-SPECT mutual features appears to be mostly unaffected by the size of the kernel window. Larger kernel window sizes (e.g., NN = 7) appear to over-smooth the two smallest SPECT-unique features (spheres 4 and 6), resulting in lower CRC, yet also achieved the highest CRC for the largest SPECT-unique feature (sphere 2–28 mm diameter).

For each of the SPECTRE reconstructions, the SNR in all six spheres and the RMSE in the three largest spheres, are shown in Figures 5A,B, respectively. For the SPECT update intensity weight (*σ*_s_) a value of 1 produced the highest SNR in SPECT-unique features. For PET-SPECT mutual features *σ*_s_ values of 1 and 5 produced a similar SNR. The lowest *σ*_s_ value investigated (*σ*_s_ = 0.1) produced the lowest SNR across all six spheres due to the higher noise present in the background compartment (see Table 2). The lowest RMSE is achieved with a *σ*_s_ value of 5, which is most evident in sphere 2 (SPECT-unique). For the investigated PET-prior intensity weights (*σ*_p_) the sphere SNR appears to be mostly unaffected. For PET-SPECT mutual features, a *σ*_p_ value of 5 produced significantly lower RMSE over *σ*_p_ values of 0.1 and 1 for the SPECTRE reconstructions. For SPECT-unique features, *σ*_p_ does not appear to have a significant influence on the RMSE. Larger distance weights (higher values of *σ*_dp_ and *σ*_ds_) resulted in improved SNR and RMSE for both mutual and unique features. Larger kernel windows produce higher SNR for both PET-SPECT mutual features and SPECT-unique features, except for the smallest sphere (10 mm diameter). The use of larger kernel windows also resulted in lower RMSE for both PET-SPECT mutual features and SPECT-unique features, with the improvement being more drastic for the SPECT-unique feature (sphere 2, 28 mm diameter). As a result, the “optimal PET-prior investigation” used parameters *σ*_p_ = *σ*_s_ = 5, *σ*_dp_ = *σ*_ds_ = 5 and NN = 5. The kernel window size (NN = 5) was chosen due to its clinical practicality over the use of larger kernel windows (lower computation time and is less susceptible to SPECT-unique feature suppression).

### OSEM_RM and SPECTRE reconstructions using the optimal PET-prior

**Figure 6:**
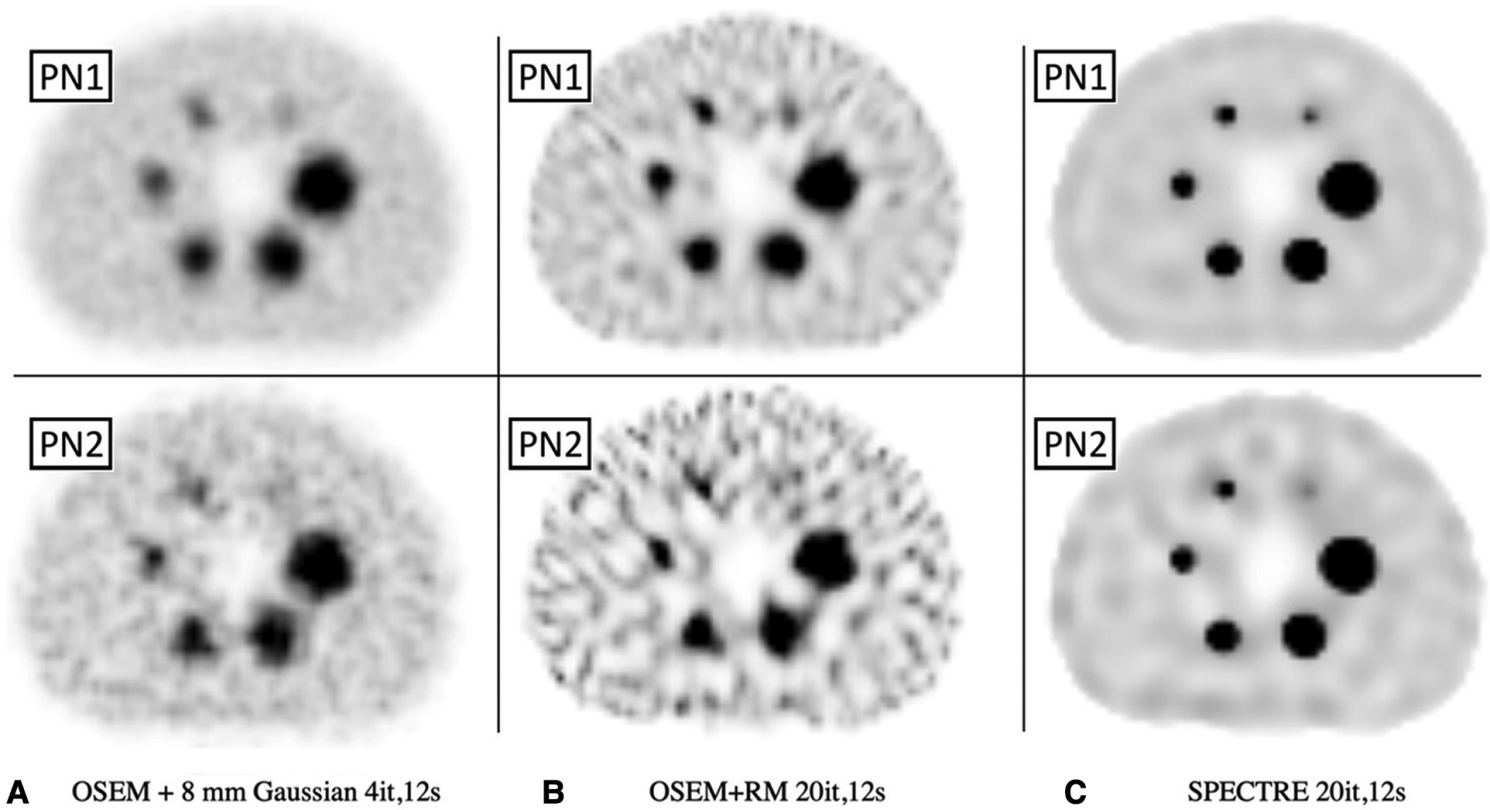
Reconstructed SPECT images of the low-noise PN1 (top row) and high noise PN2 (bottom row) 2D simulated SPECT data showing the same central axial slice for SPECT reconstructions: (**A**) OSEM std (4it) with an 8 mm Gaussian filter, (**B**) OSEM_RM, and (**C**) SPECTRE (*σ*_p_ = *σ*_s_ = 5, *σ*_dp_ = *σ*_ds_ = 5, NN = 5) reconstructions, both with 20it and 2D RM (using ^177^Lu CDRF). The same window has been applied to each series.

**Figure 7:**
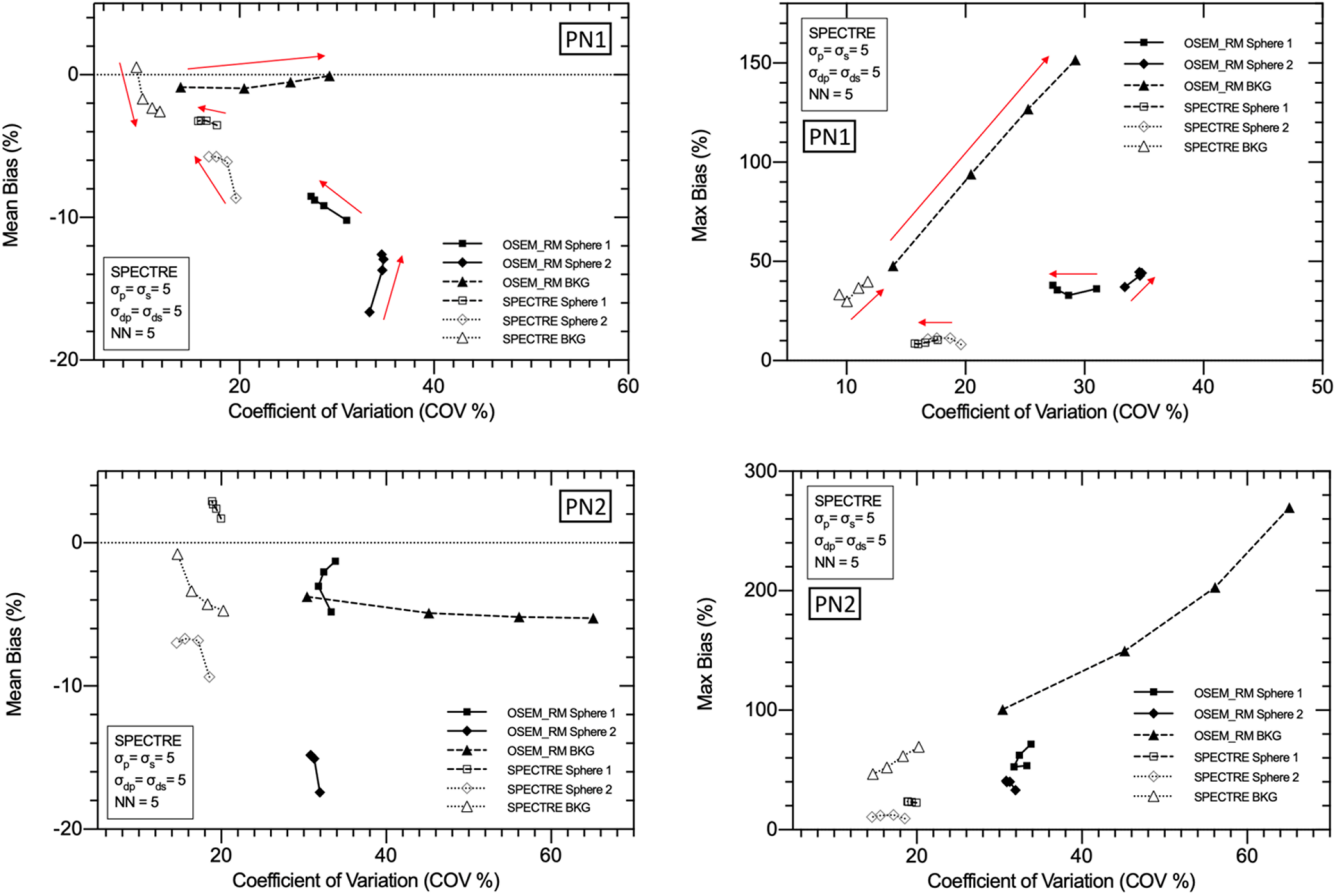
Mean bias (left) and max bias (right) vs. COV % for OSEM_RM and SPECTRE (*σ*_p_ = *σ*_s_ = 5, *σ*_dp_ = *σ*_ds_ = 5, NN = 5) reconstructions of the 2D low noise PN1 (top row), and high noise PN2 (bottom row), simulated IEC phantom data. Results for three VOIs are shown: the 37 mm and 28 mm spheres, and background compartments, for 5, 10, 15, and 20 iterations. Red arrows used to show increasing iterations.

Standard OSEM reconstructions and SPECTRE reconstructions of the PN1 (low noise) and the PN2 (high noise) 2D-simulated SPECT projection data are shown in Figure 6, and a plot of the mean (left) and maximum (right) bias vs. COV % in the two largest spheres and background compartments for the OSEM_RM and SPECTRE reconstructions are shown in Figure 7.

**Figure 8:**
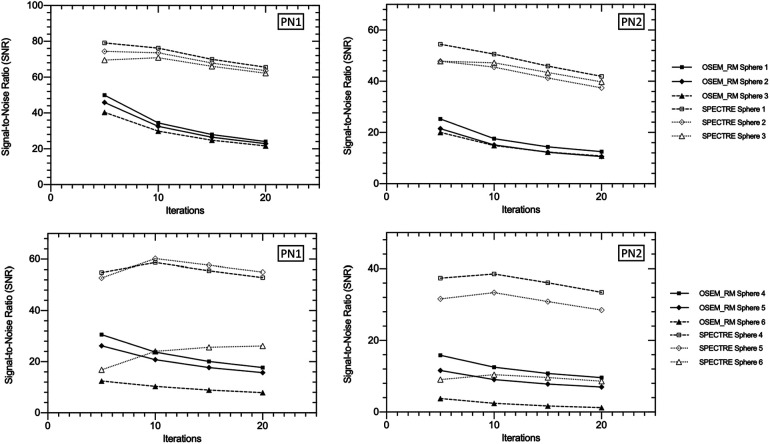
Signal-to-noise ratio (SNR) vs. number of iterations in the three largest (top row) and three smallest (bottom row) IEC phantom spheres for OSEM_RM and SPECTRE (*σ*_p_ = *σ*_s_ = 5, *σ*_dp_ = *σ*_ds_ = 5, NN = 5) reconstructions of the 2D-simulated low noise PN1 (left column), and high noise PN2 (right column), SPECT projection data.

In Figure 6, the visual improvement in the SPECTRE images is evident. This is supported in Figure 7, which shows that the SPECTRE reconstructions have improved bias and COV % in the two largest spheres when compared to the OSEM_RM reconstructed images. The COV % and maximum bias in the background compartment ROI also show improved noise characteristics for the SPECTRE reconstructions (with lower COV % and single maximum pixel bias compared to OSEM_RM). Figure 8 shows, for both the PN1 and PN2 data sets, that the SPECTRE reconstructions have a higher SNR compared to OSEM_RM for each sphere at all iterations. Using the PN1 data, the average sphere SNR for SPECTRE (54.2) shows a factor of 2.96 improvement over the OSEM_RM (18.3) at the last (20th) iteration. With the PN2 data, the average improvement in SNR of SPECTRE over OSEM_RM is even more pronounced (∼6×). However, the SNR for both the OSEM_RM and SPECTRE reconstructions is significantly lower than the PN1 example for the smallest sphere (sphere 6, 10 mm).

**Figure 9:**
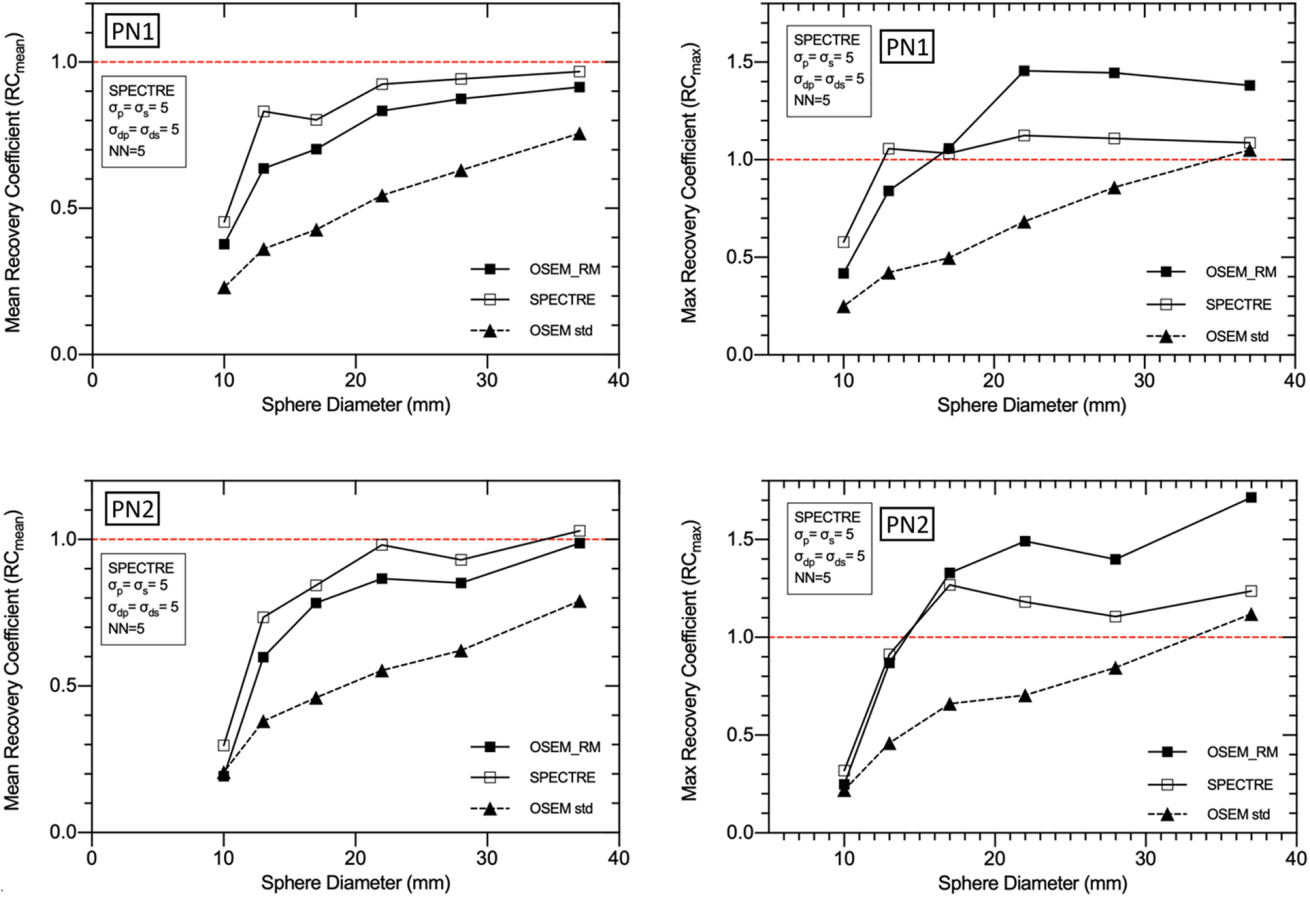
Mean (left) and maximum (right) recovery coefficients for each of the IEC phantom spheres for each reconstruction of the low noise PN1 (top row), and high noise PN2 (bottom row), 2D simulated data. Three reconstructions are shown: standard OSEM (4it12s) with a Gaussian filter of 8 mm FWHM (OSEM std), OSEM_RM, and SPECTRE (*σ*_p_ = *σ*_s_ = 5, *σ*_dp_ = *σ*_ds_ = 5, NN = 5), both using 20 iterations and 2D ^177^Lu CDR compensation.

The mean and maximum recovery coefficients in each sphere for OSEM std, OSEM_RM, and SPECTRE reconstructions are shown in Figure 9. The plot shows that the SPECTRE reconstructions of both the PN1 and PN2 data have better recovery of the true radioactivity concentration in all six spheres compared to the conventional OSEM reconstructions. The plots also show that SPECTRE reconstruction has better maximum (single voxel) recovery when compared to OSEM_RM, which shows significant overshoots of the true radioactivity concentration in the three largest spheres.

### 2D and 3D reconstructions using OSEM_RM and SPECTRE

**Figure 10:**
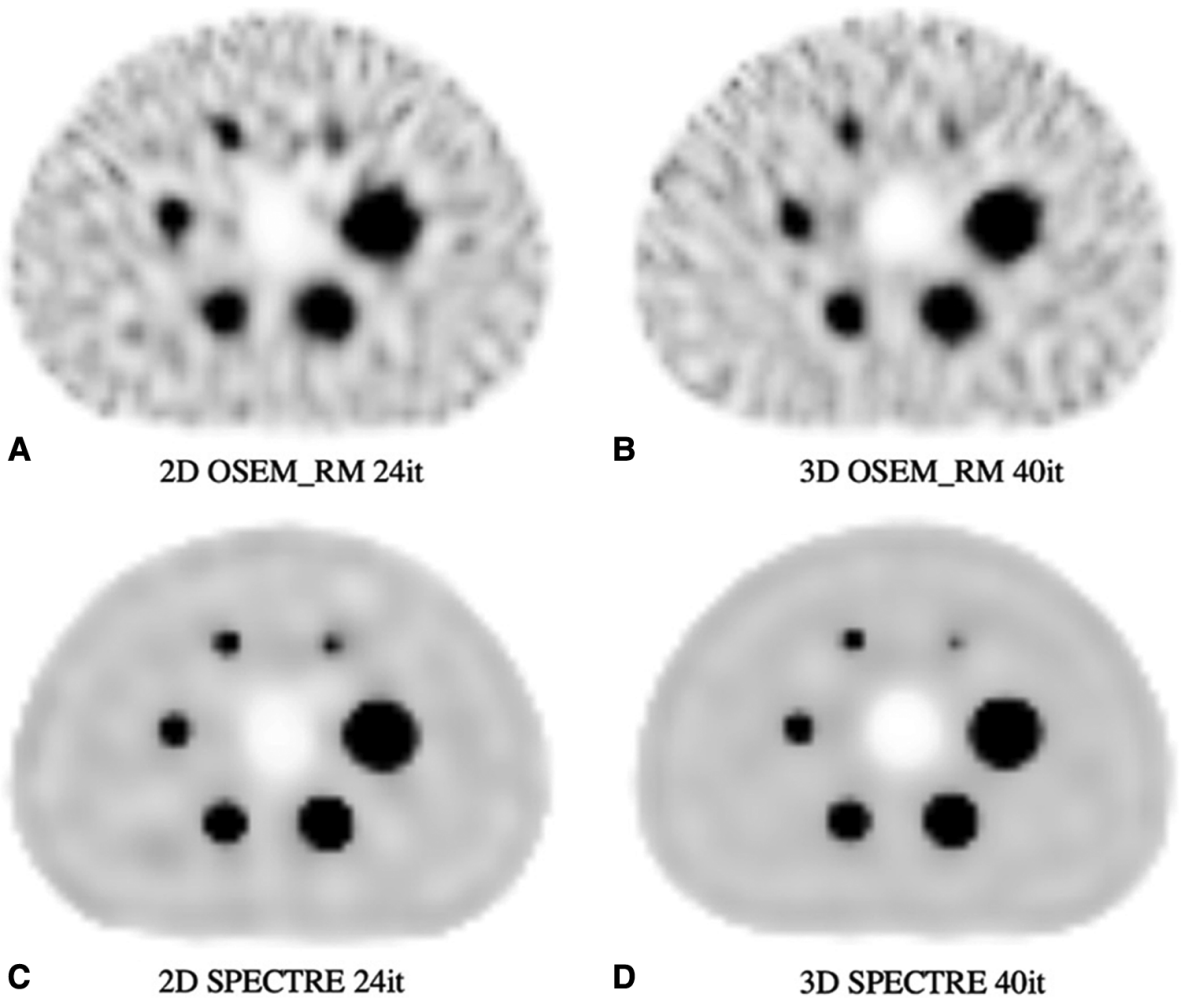
Reconstructed SPECT images of the PN1 (low noise) 2D and 3D simulated SPECT projections showing the same central axial slice: (**A**) 2D OSEM_RM for 24 iterations, (**B**) 3D OSEM_RM for 40 iterations, (**C**) 2D SPECTRE for 24 iterations, and (**D**) 3D SPECTRE for 40 iterations. SPECTRE reconstructions used the “optimal” PET-prior and HKEM parameters *σ*_p_ = 5, *σ*_s_ = 2, *σ*_dp_ = *σ*_ds_ = 15, NN = 5.

**Figure 11:**
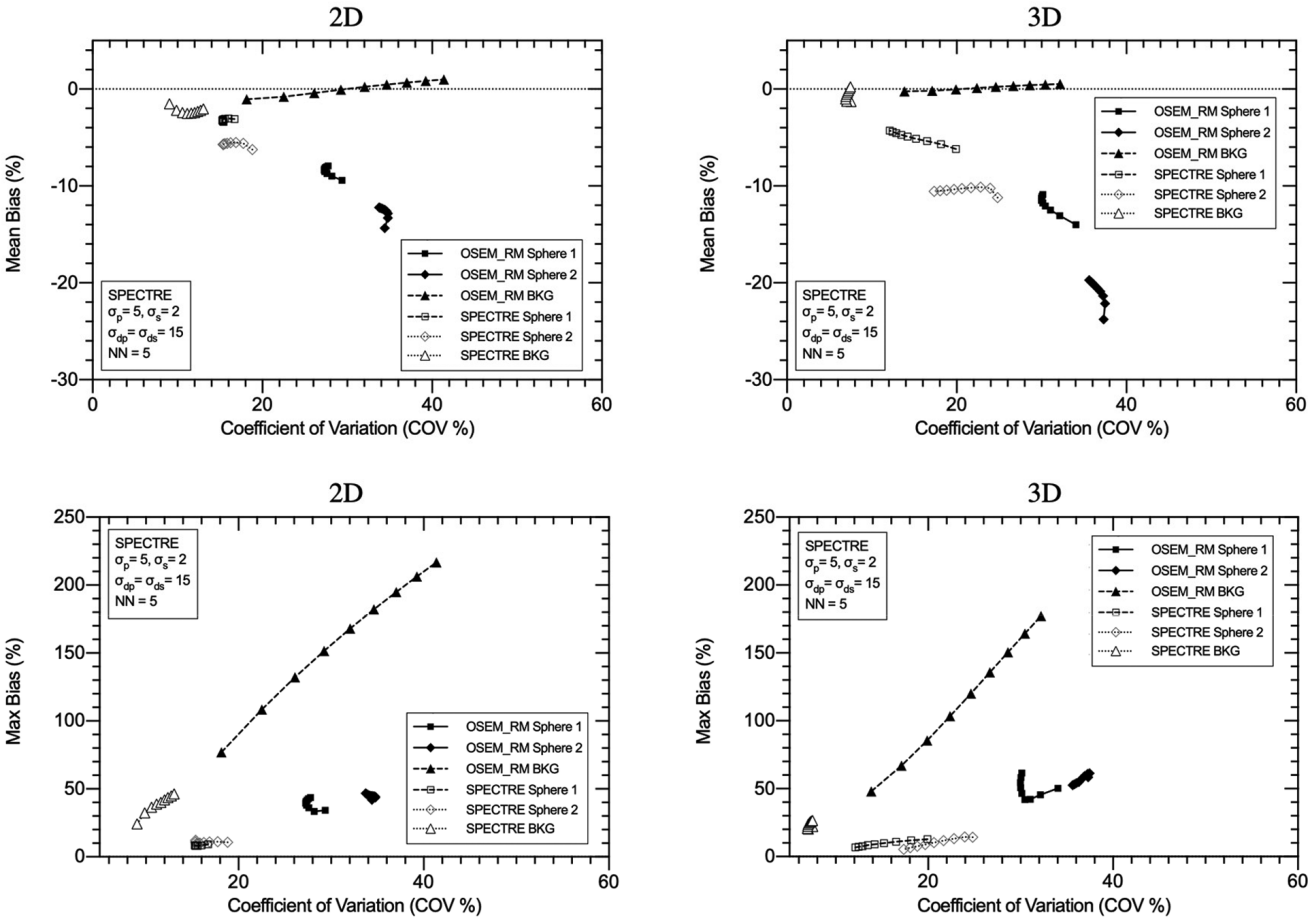
Mean bias (top row) and maximum bias (bottom row) vs. the coefficient of variation (COV %) for OSEM_RM and SPECTRE (*σ*_p_ = 5, *σ*_s_ = 2, *σ*_dp_ = *σ*_ds_ = 15, NN = 5) reconstructions of the 2D (left column) and 3D (right column) low noise simulated IEC phantom data. Three VOIs are shown in each plot: the 37 mm sphere, the 28 mm sphere, and the background compartment. Data is shown from 8 to 40 iterations in increments of four iterations between data points (9 different reconstructions for each series in total).

The optimal SPECTRE reconstruction parameters derived from the 2D investigations translated to similar quantitative improvements when applied to the 3D case, and the SPECTRE reconstructed images showed improved quantification in terms of recovery and uniformity over conventional reconstructions. OSEM_RM and SPECTRE reconstructions performed in 2D and 3D were qualitatively similar, and the quantitative improvements afforded by the SPECTRE reconstruction approach over OSEM_RM were also similar. In Figure 10, like Figure 6, the visual improvement in the SPECTRE images is striking. This figure shows the center slice of OSEM_RM and SPECTRE reconstructions of the 2D (24th iteration) and 3D (40th iteration) PN1 simulated data, with the iterations chosen based on similar background noise for OSEM_RM. However, the SPECTRE reconstructions background noise at 2D 24it and 3D 40it is not as closely matched. This is shown in Figure 11, where the mean and maximum bias vs the COV % for the two largest spheres and background compartment are compared with 2D on the left and 3D on the right. In this figure, the COV % in the background compartment for the 2D and 3D SPECTRE reconstructions have no overlap, unlike the OSEM_RM reconstructions that have similar background COV % at 2D 24it and 3D 40it.

**Figure 12:**
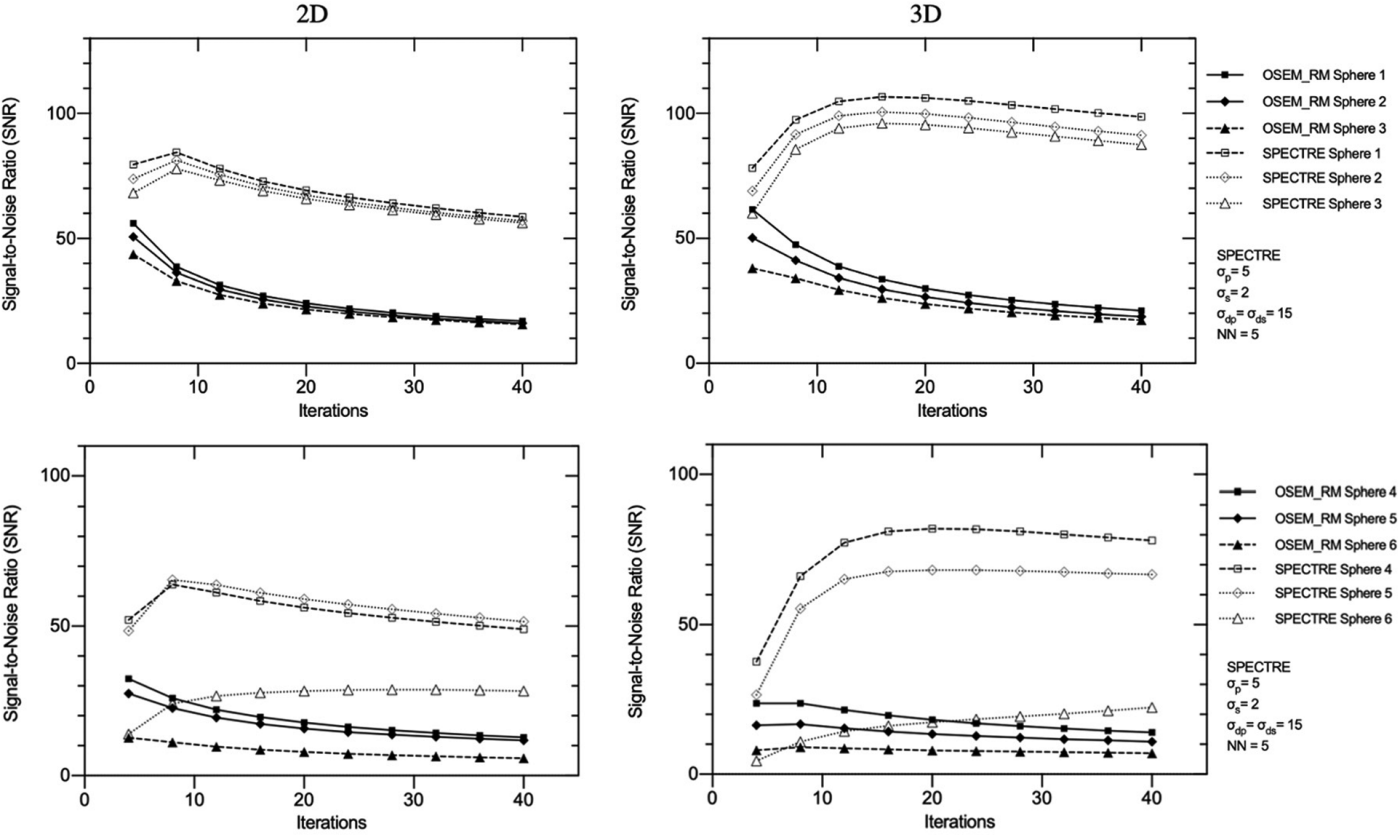
Signal-to-noise ratio (SNR) vs. number of iterations in the three largest (top) and three smallest (bottom) IEC phantom spheres for OSEM_RM and SPECTRE (*σ*_p_ = 5, *σ*_s_ = 2, *σ*_dp_ = *σ*_ds_ = 15, NN = 5) reconstructions of the PN1 low noise 2D (left) and 3D (right) simulated SPECT projection data. Data is shown from 4 to 40 iterations in increments of 4 iterations between data points (10 different reconstructions for each series in total).

SPECTRE shows an average reduction in noise (background COV %) compared to OSEM_RM by a factor of approx. 2.7 and 3.3 over 8–40 iterations for the 2D case and 3D case, respectively. Mean bias in the two largest spheres had similar improvements in quantitative accuracy in the 2D and 3D cases when comparing SPECTRE to OSEM_RM; for the images shown in Figure 10, the mean bias (in the 2D case) was reduced by a factor of 2.6 and 2.2 for spheres 1 and 2 respectively, and (in the 3D case) by a factor of 2.5 and 1.9 for spheres 1 and 2, respectively. The SNR for each sphere in the 2D and 3D SPECTRE reconstructions of the PN1 data using HKEM parameters *σ*_p_ = 5, *σ*_s_ = 2, *σ*_dp_ = *σ*_ds_ = 15, NN = 5, are shown in Figure 12. As with COV %, the SNR in the 2D and 3D OSEM_RM reconstructions appears to be more congruent than for the 2D and 3D SPECTRE reconstructions. In general, the sphere SNR for the 3D reconstructions is higher than the 2D reconstructions. The average improvement in sphere SNR across all iterations was 2.8 and 3.4 for the 2D and 3D case, respectively. Lower SNR in the 2D compared to 3D images is largely a result of relatively higher noise (COV %) in the background compartment and this difference is more exaggerated in the SNR between the 2D and 3D SPECTRE images.

**Figure 13:**
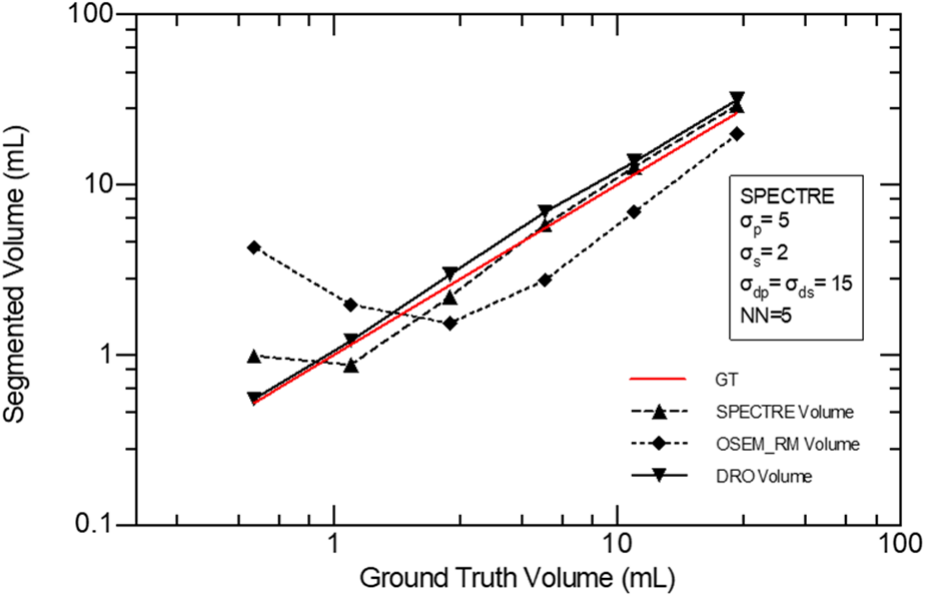
Accuracy of volume segmentation using a 42% FT segmentation method for the GT DRO, OSEM_RM, and SPECTRE reconstructed images (20 iterations).

In Figure 13, using images reconstructed with both OSEM_RM and SPECTRE (40it12s), the segmentation accuracy of the regions of elevated activity was evaluated using a 42% fixed threshold. The average % volume difference from ground truth for the OSEM_RM and SPECTRE reconstructions is 158% and 26%, respectively. Demonstrating that the SPECTRE reconstructions have significantly improved segmentation accuracy across all sphere sizes when using the 42% FT method.

## Discussion

The investigations presented in this work further demonstrate that the SPECTRE reconstruction approach shows promise for improving SPECT image quality and RNT dosimetry estimates. The SPECTRE-reconstructed images consistently showed improved quantification in terms of recovery and uniformity over the conventional reconstruction methods, and reconstructions performed in 2D closely resembled the 3D case for both OSEM_RM and SPECTRE reconstructions, both qualitatively and in terms of quantitative improvements. Each of the following discussion sections pertains to each investigation and follows the same order presented in the methods and results sections.

### SPECTRE reconstructions with missing PET support

The “lesion-less” PET-prior investigation highlighted several important insights into the HKEM algorithm when applied to the SPECTRE reconstruction approach:

(i) Distance weights that promote features closer to the central voxel (i.e., smaller *σ*_dp_ and *σ*_ds_ values) produce noisier images.
(ii) A smaller kernel window size may not sufficiently suppress noise.
(iii) Small *σ*_s_ values also contribute to high background COV %, where in the reconstruction updates, small intensity differences due to noise act as features in the SPECT update image kernel $k_{s\;f\;j}^{\left(n\right)}$ and are propagated into the resulting SPECTRE reconstructed image.

It should be noted that in this study, because the PET-prior was based on a noiseless image, small PET-prior intensity weights (*σ*_p_ = 0.1) promoted smoothing. However, this result should be treated with caution because small PET-prior intensity weights are edge-preserving with noisy PET images, this may not be the case. Conversely, if *σ*_p_ is too large, fainter structures and/or low-intensity edges present in the PET-prior might not add sufficient support to the SPECTRE reconstruction. This is evident in the smallest sphere (10 mm diameter), which suffers from large partial volume losses. A few important, albeit expected, findings were revealed:

(i) Larger kernel windows can over smooth SPECT-unique features if the window is larger than the structure.
(ii) The use of larger kernel windows and larger distance weights produces improved noise properties in the background compartment and shows improved recovery in PET-SPECT mutual features.
(iii) Higher SPECT update image intensity weights (smaller *σ*_s_ values), smaller distance weights (*σ*_ds_ & *σ*_dp_), and smaller kernel windows, all contribute to improved SPECT-unique feature recovery.

Similar observations have been made in studies using the kernel method to reconstruct PET data using MR side information; Hutchroft et al. found that the use of larger kernel windows yielded better performance for mutual MR-PET features (37); Deidda et al. demonstrated that relaxed distance weights (larger Euclidean distance weights) produced improved noise characteristics for reconstructions of low-count PET data (12, 38). A less obvious finding from this investigation was that relaxed PET-prior intensity weights (high *σ*_p_ values, low edge preservation) and relaxed distance weights (large *σ*_ds_ and *σ*_dp_ values) can produce improved recovery in PET-SPECT mutual features. This is likely due to the simulated PET image having worse spatial resolution than anatomical priors (e.g., CT and MR) that are more commonly used in guided reconstructions.

### OSEM_RM and SPECTRE reconstructions using the optimal PET-prior

Because image reconstruction is fundamentally a deblurring/denoising problem, contrast recovery improves as more iterations are performed (Figure 8). However, in the case of OSEM_RM (where a denoising mechanism is absent), amplification of noise dominates; hence the noise increases at a rate faster than the improvement in contrast recovery, leading to poorer SNR. In contrast to this, SPECTRE reconstruction shows comparatively lower noise amplification at higher iterations and the COV % in the background compartment for the SPECTRE reconstructions remains relatively constant as more iterations are performed. As a result, with each iteration the SNR in the five largest spheres for the SPECTRE reconstruction does not decrease as quickly as OSEM_RM, allowing for additional iterations to be performed for improved recovery and SNR in the smallest sphere. Improved recovery and SNR in SPECTRE reconstructions also manifest as improved mean and maximum recovery coefficients when compared to OSEM_RM (Figure 9). SPECTRE reconstructions have a maximum (single voxel) recovery that does not overshoot the true value as much as OSEM_RM; this is important as SUV metrics become increasingly adopted in quantitative SPECT imaging, where SUV_max_ is a commonly reported statistic (48, 49). The relative improvement SPECTRE sees over OSEM_RM is most evident with the low count data, both in terms of quantitative accuracy (improved recovery) and improved noise characteristics.

### 2D and 3D reconstructions using OSEM_RM and SPECTRE

To make studies of this type computationally feasible, it is desirable to use 2D simulations, which drastically reduce the computational time compared to 3D. Both the 2D and 3D reconstructions used masks to reduce the number of voxels considered in the calculation; for the 3D reconstructions, the mask reduced the number of voxels by ∼80%, significantly reducing the reconstruction time. For example, in this study, a typical 2D OSEM_RM reconstruction requires ∼110 s to compute 40 iterations compared to 4.6 h for 3D; faster by a factor of ∼150. For 2D SPECTRE, reconstructions took approximately 296 s to compute 40 iterations for NN = 5, whereas 3D SPECTRE required ∼5 h. Hence, it is important to show correspondence between the 2D and 3D reconstructions and their parameters.

The OSEM_RM and SPECTRE reconstructions translated well from 2D to 3D. The primary difference between the 2D and 3D SPECTRE reconstruction metrics resulted from the differences between their background noise, whereas superior SNR in the 3D SPECTRE reconstructions resulted the improved noise characteristics and not improved sphere recovery. We have identified two factors that are consistent with our observations. The first is the difference in the use of 2D and 3D CDR, where in the case of 2D CDR, fewer voxels, and thus fewer counts, contribute to the individual voxel update estimate, leading to higher noise (50–54). The second factor (unique to SPECTRE) is a result of the 2D vs. 3D kernel window (5 × 5 for 2D reconstructions, and 5 × 5 × 5 for 3D reconstructions); the 2D kernel window contains fewer voxels for the comparison and thus appears to suppress noise less efficiently compared to the 3D case (likely due to more voxels being considered in the 3D case, leading to better noise averaging). Despite the differences between the 2D and 3D SPECTRE reconstructed images, it is apparent that the 2D exploration and optimization approach provided valuable insights.

Finally, we looked at segmentation performance. This study was conceived from a theranostic point of view, where SPECT reconstructed images are used to estimate the adsorbed radiation dose delivered to a volume of interest. Radionuclide therapy dosimetry estimates often report on the mean absorbed dose delivered to a volume, and thus the segmentation method and image characteristics are significant contributing factors to the accuracy of the image-based absorbed dose estimates. This is particularly important for lesion dosimetry, where, unlike organ dosimetry, there often is no anatomically discernible boundary to help with segmentation (e.g., coming from the accompanying CT). Instead, segmentation generally has to rely solely on the reconstructed SPECT image itself, and currently there is no consensus on the best way to approach this with the current limitations on SPECT imaging of RNT. This task is difficult, and depends on the spatial resolution, image reconstruction algorithm and any post-filtering, size and shape of the volume, and noise/noise-related artifacts (55, 56). While improving noise properties, such as the sphere SNR, indirectly contribute to improved accuracy of absorbed dose estimates, the improved morphological properties provided by the PET-prior improve segmentation and concordance with ground truth. The SPECTRE reconstructions have significantly improved segmentation accuracy across all sphere sizes when using the 42% FT method, suggesting that the SPECTRE reconstructions are better suited for the application of FT segmentation methods, when compared to conventional OSEM reconstructions, leading to more accurate image-based lesion dosimetry estimates.

### Limitations and general discussion

Translation of HKEM parameters used in this investigation to clinical patient data should be done with care. Parameters that show improved SNR for simulated data may not produce reconstructed images that have improved task performance. For instance, for improved RNT dosimetry estimates, better RC_mean_, RC_max_, and lower RMSE might be better metrics to optimise as they may correlate more strongly with segmentation and dosimetry task performance. Likewise, for SPECT-unique features, larger kernel windows can reduce the recovery even if the SNR is improved. This highlights that each of the metrics presented in this work needs to be considered in parallel, and that optimization depends on the imaging task at hand.

In contrast to previous studies of HKEM, where MR side information was the guiding modality, this study implicitly assumes that PET-SPECT features, or indeed radionuclide distribution in a theranostic setting, are largely similar (this is true even for the lesions-less images, where only limited regions of the phantoms differed). The implementation of PET images as *a-priori* information for guiding SPECT reconstructions presents new challenges. For objects like the IEC phantom, where similarities between the PET-prior and SPECT projection data are expected, larger kernel windows and more relaxed distance weights will yield better results (37). Furthermore, in clinical implementations of SPECTRE, where differences between the PET and SPECT data are expected (and co-registration of the PET/SPECT images is challenging), the use of larger kernel windows and larger Euclidean distance weights may lead to the suppression of SPECT-unique features. This is possible even for perfectly co-registered PET-SPECT data if certain HKEM parameters are used (i.e., parameters that promote smoothness, such as high *σ*_p_ and large distance weights, in conjunction with large kernel windows). We add that the PET and SPECT radionuclide distribution functions are dependent on the post-administration imaging time and may differ due to one modality being imaged later in its distribution time course. Future investigations of the SPECTRE reconstruction approach should investigate this and optimise the HKEM parameters using a noisy PET-prior to see how its noise impacts the SPECTRE reconstructed images. Another potential issue is the time delay between diagnostic PET imaging and therapy, where changes in patient morphology and progression of the disease could lead to lower concordance between mutual information shared in the SPECT data and guiding PET images. We do not expect this to be an issue for most patients, but if disease progression is expected then another PET scan could be performed prior to therapy. The success of SPECTRE also relies on accurate co-registration between the PET image and SPECT data; future clinical investigations looking at this reconstruction approach should aim to minimize these factors by taking care of patient positioning and administration protocols.

## Conclusions

To the best of our knowledge this is the first in-depth exploration of PET-guided SPECT reconstructions, and as such, it reveals several insights that need to be considered when using the SPECTRE reconstruction approach. This work expands upon our previous investigation where we first demonstrated this reconstruction approach. Exploration and optimization of the SPECTRE approach benefited from both fast analytical SPECT simulations and faster SPECTRE reconstruction times afforded by first considering the 2D case. The impact of HKEM parameters for both PET-SPECT mutual features and SPECT-unique features was investigated using simulated data from the NEMA IEC body phantom test object. Comparisons were made to conventional OSEM_RM reconstructions using metrics such as mean and maximum bias, COV %, sphere recovery coefficients, sphere SNR, and RMSE. The improvement in SPECT image quality over conventional reconstruction methods was demonstrated in the context of accurate radionuclide therapy absorbed dose estimates. A novel accelerated algorithm exploration approach was also investigated whereby simulations and reconstructions were first performed in 2D and then applied to the 3D case, and the feasibility of this approach was validated by comparing results from the two. This investigation into the SPECTRE reconstruction approach further demonstrates a significant potential for improved SPECT image quality, leading to more accurate SPECT-based lesion dosimetry estimates when conventional segmentation methods are used.

## Data Availability

The data supporting the conclusions of this article can be made available upon request.
